# InsuLock: A Weakly Supervised Learning Approach for Accurate Insulator Prediction, and Variant Impact Quantification

**DOI:** 10.3390/genes13040621

**Published:** 2022-03-30

**Authors:** Shushrruth Sai Srinivasan, Yanwen Gong, Siwei Xu, Ahyeon Hwang, Min Xu, Matthew J. Girgenti, Jing Zhang

**Affiliations:** 1 Computer Science Department, University of California, Irvine, CA 92697, USA; shushrruthsai@gmail.com (S.S.S.); siweix3@uci.edu (S.X.); 2 Mathematical, Computational & Systems Biology, University of California, Irvine, CA 92697, USA; yanweng@uci.edu (Y.G.); ahyeon.hwang@uci.edu (A.H.); 3 Computational Biology Department, School of Computer Science, Carnegie Mellon University, Pittsburgh, PA 15213, USA; mxu1@cs.cmu.edu; 4 Department of Psychiatry, School of Medicine, Yale University, New Haven, CT 06520, USA; matthew.girgenti@yale.edu

**Keywords:** 3D chromatin structure, *CTCF* mediated insulator loops, gene regulation, deep learning, brain disorders

## Abstract

Mapping chromatin insulator loops is crucial to investigating genome evolution, elucidating critical biological functions, and ultimately quantifying variant impact in diseases. However, chromatin conformation profiling assays are usually expensive, time-consuming, and may report fuzzy insulator annotations with low resolution. Therefore, we propose a weakly supervised deep learning method, InsuLock, to address these challenges. Specifically, InsuLock first utilizes a Siamese neural network to predict the existence of insulators within a given region (up to 2000 bp). Then, it uses an object detection module for precise insulator boundary localization via gradient-weighted class activation mapping (~40 bp resolution). Finally, it quantifies variant impacts by comparing the insulator score differences between the wild-type and mutant alleles. We applied InsuLock on various bulk and single-cell datasets for performance testing and benchmarking. We showed that it outperformed existing methods with an AUROC of ~0.96 and condensed insulator annotations to ~2.5% of their original size while still demonstrating higher conservation scores and better motif enrichments. Finally, we utilized InsuLock to make cell-type-specific variant impacts from brain scATAC-seq data and identified a schizophrenia GWAS variant disrupting an insulator loop proximal to a known risk gene, indicating a possible new mechanism of action for the disease.

## 1. Introduction

The human genome is hierarchically compacted and organized in the three-dimensional space (3D), including large chromosomal domains, nuclear compartments, topological associated domains (TADs), and chromatin loops. The dynamic nature of chromosome conformations and three-dimensional genome organizations leads to a variety of chromatin interactions, which play essential roles in critical biological processes such as transcription, proliferation, homeostasis, division, and even disease mechanisms [1,2,3]. Several chromatin conformation capture assays, such as Hi-C and ChIA-PET [4,5], have revealed that chromatin loops are often mediated by cohesin, a multi-component ring-shaped protein, and an insulator protein called *CTCF* (11-zinc finger protein) [6,7]. The highly studied loop extrusion model suggests that cohesin extrudes chromatin to form loops anchored at *CTCF* sites, that are called chromatin insulator loops. Lines of evidence have shown that genetic variations at insulator loop anchors may cause significant genome organizational changes, subsequently introducing aberrant promoter–enhancer interactions in the affected TADs, resulting in a variety of human genetic disorders [8,9,10,11,12,13,14,15,16,17,18,19]. Thus, insulator discovery and characterization are pivotal to understanding gene regulation and disease mechanisms.

To date, several computational methods have been proposed to predict chromatin loops. For instance, Kai et al. proposed a computational method called Lollipop to predict chromatin loops from a set of genomic and epigenomic features using a random forest classifier [20]. However, Lollipop does not utilize DNA sequence information and therefore cannot directly quantify variant impacts at chromatin loop anchors. CTCF-MP is another method that utilizes genomic and epigenomics features to make *CTCF*-mediated loop predictions [21]. It employs a word2vec model to encode DNA sequences into feature vectors and then combines other epigenomic features for loop predictions using a boosted tree classifier. While CTCF-MP demonstrated improved performance, its ability to account for variant impacts using word2vec is quite limited. Recently, sequence-based deep learning methods have shown great potential for predicting insulators and quantifying variant impact therein [22,23,24]. However, the input sequences for these models are usually up to a kilobase pair in length, which is an order longer than the true insulator sites. Hence, a daunting question remains: where is the insulator boundary given a long sequence? In addition, recent studies have pointed out the involvement of novel, cell-type-specific transcription factors (TFs) other than *CTCF*, such as *ZNF143* [25], *YY1* [26], and *CUX1* [27], in chromatin looping. These findings warrant the need for improved computational approaches with the ability to refine current loop anchor annotations and remove redundant non-functional regions, ultimately facilitating downstream analyses, such as causal variant identification and functional validation. 

**Figure 1:**
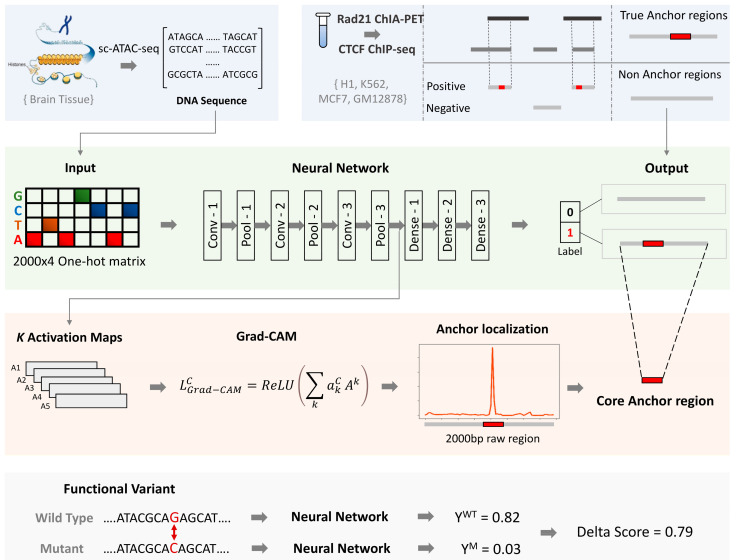
Overall schematic workflow of the InsuLock Method: First, the positive dataset is generated by intersecting *Rad21* ChIA-PET peaks with *CTCF* ChIP-seq peaks to define true anchor regions. InsuLock utilizes one-hot encoded DNA matrices as input to make predictions. Second, the binary-classification module learns the sequence patterns around the anchors of insulator loops and makes a binary prediction. Third, the weakly-supervised object detection module utilizes the activation maps (*A^k^*), weighted by an importance score (*a^c^*) to refine and localize the core anchor region and visualize the sequence features that were used by the model to make the prediction. Fourth, the variant impact quantification module identifies insulator disrupting variants by computing a delta score.

To address these challenges, we propose InsuLock, a weakly-supervised deep learning approach to predict anchors of insulator loops, localize their precise boundaries, and quantify cell-type-specific variant impacts developed within the Zhang lab at University of California, Irvine, CA, USA (Figure 1). Precisely, InsuLock constitutes three distinct modules: (i) a binary classification module based on Siamese convolutional neural network (CNN) to predict the existence of insulator within a given genomic region; (ii) a weakly supervised object detection module to refine insulator boundaries; (iii) a variant impact quantification module to prioritize variants affecting insulator functions. To test the effectiveness of InsuLock, we performed extensive benchmarking via publicly available data from ENCODE and demonstrated its high accuracy and sensitivity via cross-cell-line and leave-one-out chromosome validations. Moreover, we validated InsuLock’s object detection module’s ability to refine fuzzy insulator annotations by performing phylogenetic conservation and transcription factor (TF) motif enrichment analyses. Finally, we applied InsuLock on brain single-cell ATAC-seq data to quantify cell-type-specific impact scores of GWAS SNPs associated with Schizophrenia. In a cell-type-specific manner, we prioritized potential disease-causing variants disrupting the 3D genome organization in the human brain. 

## 2. Materials and Methods

### 2.1. Data Preparation

We collected *Rad21* ChIA-PET data along with *CTCF* ChIP-seq and ATAC-seq data on four human cell lines (H1, GM12878, K562, and MCF7) from the ENCODE web portal [28,29]. *CTCF* ChIP-seq peaks overlapping with *Rad21* ChIA-PET peaks were defined as true anchor regions and were treated as the positive samples. All the *CTCF* ChIP-seq and ATAC-seq peaks not overlapping with the true anchor regions were considered the negative samples. For ease of analyses, the negative samples were further classified into three categories (type-1, type-2, and type-3). Type-1 negatives were the *CTCF* ChIP-seq peaks that did not overlap with any true anchors, while type-2 and type-3 were the negative ATAC-seq data with and without a *CTCF* motif, respectively. We used FIMO to scan for the presence of a *CTCF* motif using the *CTCF* motif position weight matrix that was obtained from JASPAR (ID: MA0139.1, accessed on 29 January 2022) [30,31]. A threshold *p*-value of 5 × 10^−5^ was used to scan for motifs. 

Next, we randomly split our data into training, validation, and test sets in an 8:1:1 ratio, and the negative samples in the training set were manually resized to equal the number of positive training samples. All the sequences were normalized to a length of 2000 base pairs centered around their original peaks and were converted into a 2000 × 4 one-hot matrix, with each column corresponding to A, T, C, and G. 

### 2.2. InsuLock Neural Network Architecture

**Figure 2:**
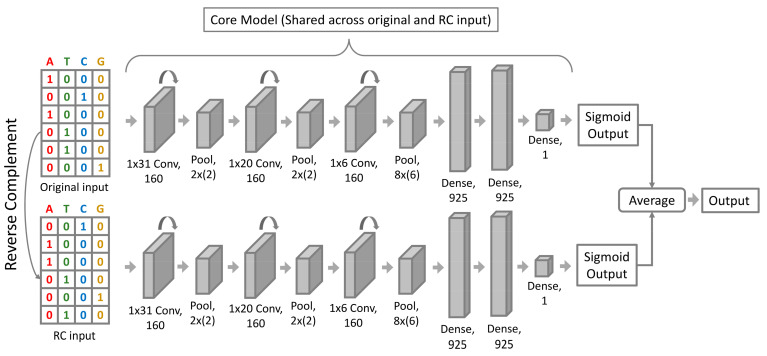
InsuLock’s Siamese neural network architecture: To the binary classification model, the one-hot encoded matrix of both the forward and reverse complementary sequences are fed, and the sigmoid predictions of both the strands are averaged out to provide the final anchor prediction. The neural network architecture is composed of 3 convolution blocks, where each block contains a convolution and a pooling layer, which is followed by 3 dense layers.

As shown in Figure 2, we adopted a Siamese/conjoined neural network architecture, consisting of convolutional, pooling, and fully connected layers. As the word Siamese suggests, there were two copies of the same convolutional neural network with shared weight parameters that run in parallel, whose outputs were combined to provide a single output. Each network consisted of three convolution blocks, where each block consisted of a convolution layer, a Rectified Linear Unit layer (ReLU), followed by a max-pooling layer. To train the network, we used a 2000 × 4 one-hot encoded DNA matrix as input. The filters of the first convolution layer had a size of 31 × 4, which represented the position weight matrices (PWM) of the motif patterns in the input sequence. Then, the max-pooling layers were used to summarize and aggregate the features captured by the convolution layers. The later convolution filters captured the higher-order DNA interactions and higher-level DNA features such as GC content. The outputs of the final max pool layer were then introduced into fully connected layers to make a sigmoid prediction on the probability of the input sequence containing an insulator loop anchor region. Fifty percent dropout was used on the fully connected layers to reduce overfitting [32].

(1) $$P(\mathrm{anchor})=1/\left(1+e^{-x}\right)$$

The rationale behind adopting a Siamese architecture was to overcome the strand duality issue that had been persistent in all predictive models that map double-stranded DNA to regulatory functions. In an ideal world, both the forward and the reverse strand must produce analogous predictions when given as input. Nevertheless, since current neural network architectures were not developed explicitly for DNA analyses but rather for computer vision tasks, they produce highly varied predictions on the forward and reverse DNA strand sequences. To overcome this issue, we trained a post-hoc conjoined model that could be described as a standard model trained with augmented reverse complementary (RC) sequences, but was converted into a conjoined model after the model training, where it took in two inputs: the forward and reverse strand sequences. The outputs of the two strands were then averaged to provide the final anchor probability. 

### 2.3. Model Training and Evaluation

Our neural network models were trained using the Adam optimization algorithm with a learning rate of 5 × 10^−5^ for 50 epochs, with a batch size of 64 to minimize the average binary cross-entropy loss on the training set [33]. A weight decay of 5 × 10^−4^ was used alongside the Adam optimizer. The performance of the model on the validation data was evaluated at the end of each epoch, and the model with the lowest loss on the validation data was saved and used.

To facilitate the training process and improve model performance, we employed a two-stage training process. In the first stage, we pre-trained a binary classifier that classified sequences based on the presence of a *CTCF* motif. In the second stage, we trained a binary classifier that predicted insulator loop anchors by initializing the network with the weights of the pre-trained model.

We performed cross-cell-line validation and cross-chromosome validation across all four cell lines and all 22 autosomal chromosomes to measure how well the model generalized to a new cell line or chromosome and also to measure the loop anchor’s conservation across different cell lines and chromosomes. For cross cell-line validation, we trained a neural network model on each of the four cell lines and tested each model on all four cell lines. For cross chromosome validation, we trained 22 models, where each model was trained on 21 chromosomes, leaving one chromosome out for validation. All the models were initialized using the weights of the pre-trained model, ensuring a robust comparison. Models with the best performance on the validation set were used to report the performance. Due to the severe class imbalance in our dataset, we used a combination of area under the precision-recall curve (AUPR) and area under the receiver operating characteristic curve (AUROC) as our primary metric to measure the performance of the models. The neural network architectures were implemented using the PyTorch machine learning library, and the training process was accelerated using the NVIDIA Tesla T4 GPU (NVIDIA Corporation, Santa Clara, CA, USA) [34].

### 2.4. Weakly Supervised Object Detection via Gradient-Weighted Class Activation Mapping

To accurately refine the insulator boundaries, we used a popular approach in computer vision named gradient-weighted class activation mapping (Grad-CAM) to visualize the predictions made by the neural network [35]. Specifically, Grad-CAM utilizes the gradient information flowing through convolution layers to calculate a scalar importance score ($a_{k}^{C}$) for each activation map (*A^k^*) through a global-average pooling operation, as shown in Equation (2).

(2) $$a_{k}^{C}=\frac{1}{z}\underset{i}{\sum }\underset{j}{\sum }\frac{\partial y^{C}}{\partial A_{ij}^{k}}\;⏟\;\mathrm{Global}\;\mathrm{Average}\;\mathrm{Pooling}$$

A weighted linear combination is then performed on the activation maps using the scalar importance scores, followed by a RELU function to produce a Grad-CAM heat map of dimensions i×j (Equation (3)), which could be mapped back to the input 2D-image to visualize the important features the model used to make the predictions.

(3) $$L_{Grad-CAM}^{C}=RELU\left(\underset{k}{\sum }a_{k}^{C}A^{k}\right)$$

Due to the 1-dimensional nature of DNA sequences, the equation for calculating the scalar importance score was modified as shown in Equation (4). A 1D Grad-CAM map was generated through a weighted linear combination of the activation maps.

(4) $$a_{k}^{C}=\frac{1}{z}\underset{i}{\sum }\frac{\partial y^{C=1}}{\partial A_{i}^{k}}$$

In our method, we used the gradients flowing through the final convolution block to produce the Grad-CAM importance map and refine the insulator boundaries. Since the sequence length after the final convolution block was shorter compared to the original input length, we used PyTorch’s inbuilt up-sampling function to resize the importance score array to match the input length. Grad-CAM was then applied to only the true insulator samples. To refine our predictions, we used a bin size of 40 base pairs around the high Grad-CAM score region to define the important regions. This method enabled us to understand the features of insulator loop anchors by visualizing and elucidating the basis of our model’s decision-making.

### 2.5. Transcription Factor Motif Enrichment Analyses

To uncover and analyze the refined insulator regions, we performed transcription factor enrichment analyses to identify TFs that play a role in chromatin looping. Specifically, we downloaded the position weight matrices (PWM) of 984 motifs in Homo sapiens from JASPAR and then used CentriMo (MEME SUITE) with default parameters to perform the motif enrichment analyses on both the forward and reverse strands of the refined insulator regions [36].

### 2.6. Phylogenetic Conservation Analyses

To test the efficiency of the weakly supervised object detection module in condensing and refining the insulator regions robustly, we performed cross-species conservation analyses using PhastCons [37]. PhastCons is a hidden Markov model trained on 100 different vertebrate species and computes the conservation of a given genomic region. We specifically downloaded the hg38 100-way PhastCons bigwig file and computed the average conservation score of both the original anchor regions and the refined anchor regions. The boxplots of the conservation scores were then plotted, and the one-sided Wilcoxon test was performed to calculate the *p*-value.

### 2.7. Variant Impact Quantification at Single-Cell Resolution

To identify disease-causing variants that alter 3D genome assembly at the single-cell level, we downloaded brain sc-ATAC-seq data from Corces et al. and schizophrenia-associated SNPs from the GWAS catalog [38]. The brain sc-ATAC-seq data consisted of six main cell types (excitatory neuron, inhibitory neurons, microglia, oligodendrocytes, astrocytes, and oligodendrocytes progenitor cells) and had a total of 221,062 peaks. We overlapped the ATAC-seq peaks with the schizophrenia-related SNPs to identify chromatin accessible regions containing disease-causing variants and generated wild-type (WT) and mutant sequences centered at the site of mutation. Then we used the pre-trained InsuLock model to calculate the anchor probability of WT and mutant sequences on both the forward and reverse strand and computed a delta score for each variant. 

## 3. Results

Distinct from traditional binary classification methods, we employed a weakly supervised object detection method (WSOD) in the form of post-hoc attention to identify insulators and refine their boundaries in the human genome. Specifically, we utilized chromatin insulators loops from ENCODE to train and test our InsuLock model. Results Section 3.1 and Section 3.2 demonstrate the ability of InsuLock to make accurate insulator predictions. Then for object detection, we utilized Grad-CAM in the form of post-hoc attention to refine insulator predictions. Results Section 3.3 and Section 3.4 describe our validation outcomes on InsuLock’s refined insulator annotations. Finally, we utilized single-cell ATAC-seq data from brain tissue to predict the impact of schizophrenia-causing SNVs identified by GWAS, as shown in Result Section 3.5. In short, we applied InsuLock to various real-world datasets for comprehensive benchmarking and demonstrated its benefits over state-of-the-art insulator prediction methods.

### 3.1. InsuLock Provides Accurate Insulator Predictions

InsuLock is a deep neural network model developed to predict anchors of insulator loops and to discover the underlying sequence patterns in these regions. First, InsuLock was trained using data from four different cell lines: GM12878, H1, K562, and MCF7. The current hypothesis behind DNA loop extrusion requires co-occupancy of *CTCF* and cohesin complex at topologically associating domains (TADs) boundaries and loop anchors. Thus, we only used the sequences containing both *CTCF* and cohesion to define our positive dataset (details in Methods Section 2.1). The positive and negative training datasets from all the cell types were merged and shuffled to train a binary classifier for predicting the presence of insulator loop anchors in any given 2000 bp genomic region.

To evaluate the performance of our model, we used three different out-of-sample testing sets that contained the same set of true anchors as the positive sample but contained different negative samples. The first negative set contained *CTCF* ChIP-seq peaks that were not anchors of any chromatin loop. The second negative set contained ATAC-seq peaks with *CTCF* motifs that did not overlap with any true anchor, while the third set contained ATAC-seq peaks without a *CTCF* motif.

**Figure 3:**
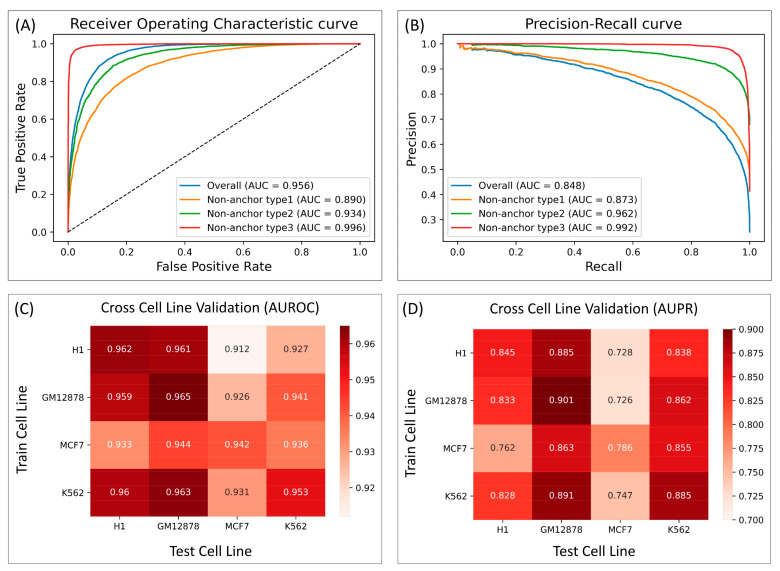
InsuLock’s performance on the test data indicating the performance metrics against different types of non-anchors using (**A**) ROC curve and (**B**) PR curve. Cross cell line validation performance against all the 4 cell lines using (**C**) AUROC and (**D**) AUPR.

As shown in Figure 3A,B, the ability of the model to differentiate between non-anchor type-3 and true anchor (AUPR = 0.992) was significantly better than that of non-anchor type-1 (AUPR = 0.873), given that many motif patterns may be shared across the *CTCF* motif-containing type-1 negatives and true anchor regions. Non-anchor type2, even though it contained *CTCF* motifs, achieved an AUPR of 0.962, which is significantly higher than that of type-1 negative, suggesting that the loop anchors were not just characterized by a single motif pattern, but rather by a combination of motif patterns, which our model was able to identify. Lastly, to test the robustness of InsuLock’s learning process across different chromosomes, we performed leave-one-chromosome-out validation and observed a consistently high AUROC and AUPR performance across all the chromosomes (average AUPR of 0.819 and average AUROC of 0.947, Figure S1).

We also performed cross-cell-line validation to test whether our InsuLock model could learn transferrable feature patterns from one cell type and make reliable predictions on new cell types not seen during the training process. As shown in Figure 3C,D, the cross cell-line performance of GM12878, H1, and K562 were consistently high (AUPR ranging from 0.828 to 0.891) compared to the merged data (AUPR = 0.848). On the other hand, the InsuLock model trained on GM12878, H1, and K562 showed slightly worse performance when predicting insulators on MCF7 (average AUPR of 0.733). Such lowered performance may be explained by the severe class imbalance in MCF7, resulting in a smaller AUPR. As demonstrated, our InsuLock model can make accurate anchor predictions and robustly transfer the learned features onto novel, unseen cell lines and loci.

### 3.2. InsuLock’s Post-Hoc Siamese Model Outperform Standard Models

To benchmark the performance of our post-hoc conjoined model, we also trained a conjoined model using the mean predictions from the forward and reverse strand during the training process to perform the back-propagation for the weights.

**Figure 4:**
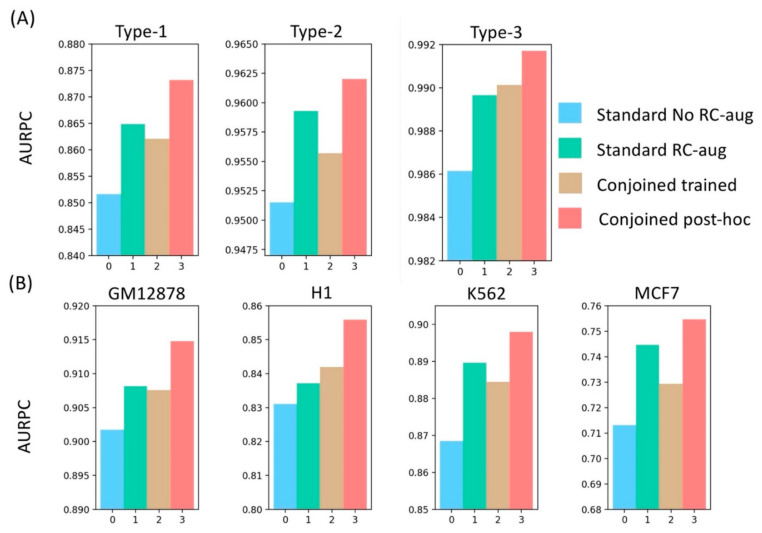
Benchmarking reverse-complement tackling strategies used in deep learning models in genomics on (**A**) different types of non-anchors and (**B**) different cell-lines using AUPR, indicating the superior performance of conjoined post-hoc models compared to other methods.

We saw that the post-hoc conjoined model consistently outperformed the trained conjoined model across all the datasets, as shown in Figure 4. This could be due to averaging of the sigmoid outputs, which might lead to inefficient back-propagation, especially in the case of divergent predictions on the forward and reverse strand. In that case, gradient descent update would negatively impact the optimization process and might lead to overfitting. On the other hand, post-hoc models learned the sequence features from both the forward and reverse strands independently and were converted to a conjoined model only after training, which was useful especially in cases where the model had to learn TF motif patterns that are non-palindromic, such as *CTCF*. Thus, it resulted in a consistent 2% better performance in all cell types. We also demonstrated that even the standard models trained with RC augmented data performed consistently better than trained-conjoined models, supporting our overfitting hypothesis of trained-conjoined models on the training data. Similarly, a previous study also showed that training both forward and reverse strands independently improved the performance of sequence-based prediction tasks [39].

### 3.3. InsuLock’s Object Detection Module Accurately Refines Insulator Boundaries That Are Highly Conserved across Species

**Figure 5:**
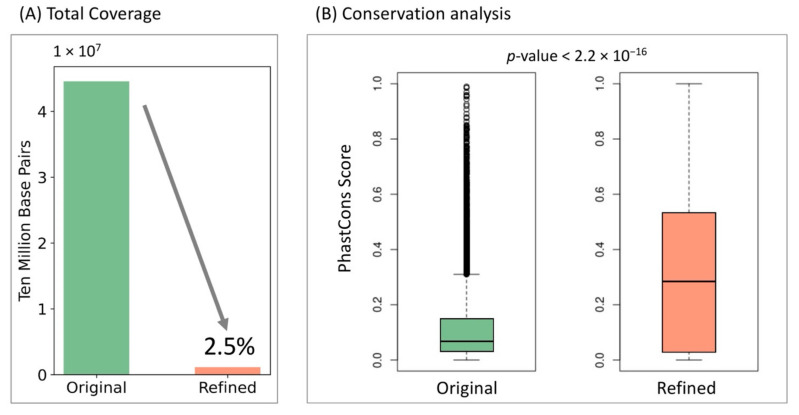
(**A**) Total nucleotide coverage of the original insulator annotations compared with InsuLock’s refined insulator annotations. (**B**) Conservation analyses using PhastCons score indicate that the refined regions are significantly conserved compared to original regions.

Scientists usually combine both chromatin accessibility information from ATAC-seq or DNase-seq and sequencing features to make insulator predictions. However, the peak calling process, especially from the recently developed single-cell ATAC-seq, usually suffers from large noise due to the extreme sparsity, resulting in relatively broad peaks up to several kilobase pairs [40,41]. Such fuzzy peak annotations further introduce inaccurate insulator boundary definitions. Here, we used a weakly supervised learning scheme to extract core features important for positive decision-making and refined core insulator boundaries by reducing the total peak coverage to only 2.5 percent of its original coverage (Figure 5A). Since cross-species conservation is a strong indicator for functionality, we compared the cross-species conservation scores of the original and refined anchor regions to test InsuLock’s ability to identify core anchor regions. Specifically, we downloaded the 100-way PhastCons signals (hg38) from the UCSC genome browser and calculated the average conservation score of all the sequences across 100 vertebrate species. We found that the refined regions were highly conserved with a median conservation score of 0.294, compared to those of the original regions that had a median score of 0.061 as shown in Figure 5B (*p*-value < 2.2 × 10^−16^, one-sided Wilcoxon test), demonstrating InsuLock’s ability to detect functional insulator regions with coarse input annotations.

### 3.4. InsuLock Facilitates Precise Motif Discoveries to Uncover Novel TF Involvement to form Insulators

To understand the features present around the loop anchor region, we used 52,495 true anchors from all four cell lines. We performed Grad-CAM on these positive samples to generate heatmaps that identified regions within these sequences that were considered important by the predicting model. For each sequence, the 40 bp window centered around the region with the highest Grad-CAM score was used to perform the motif enrichment analyses.

Then we downloaded the PWMs of 984 motifs in Homo sapiens from JASPAR. We then used CentriMo (MEME SUITE) with default parameters to perform the motif enrichment analyses on both the forward and reverse strands of the refined insulator regions. As expected, we found that *CTCF* showed the highest motif-enrichment ($-\log\left(p\right)=4065$), consistent with the well-known role of *CTCF* as an insulator. Moreover, motifs of nuclear receptor TFs such as *NR2C1* and *NR2C2*, and the motif of *YY2* were highly enriched at insulator sites. Although there has been no proof for their involvement in a chromatin loop formation, *YY1*, the paralog of *YY2*, has been known to instigate loop formation. In addition, we also identified the presence of several other motifs from InsuLock’s refined predictions, including the significant enrichment of zinc-finger transcription factors (ZF-TFs) such as *ZIC4* and *ZIC1* that are known to be enriched at anchors of *CTCF* loops [42], and *ZNF384* that provides sequence specificity to cohesin mediated chromatin interaction [43]. The motif pattern of *TCF3* that is known to be centered at loop anchor regions was also identified by our method [44]. We also identified several novel TFs whose role in TAD formation may be previously underappreciated, including *CUX1*, *SPI1*, *E2F3*, *RUNX3*, *EBF1*, *MAX*, *TFAP2C*, and *JUND* [27,43]. The detailed list of TFs identified by InsuLock can be found at Tables S1 and S2.

### 3.5. InsuLock Predicts Anchor Disruption Due to Functional Disease-Associated SNPs

The majority of the disease-causing mutations identified by GWAS lie within the non-coding regions such as enhancers, promoters, and insulators [45]. Recent studies have shown that disruptions in loop boundaries lead to aberrant enhancer–gene linkages and, consequently, altered gene expression profiles in genetic disorders [25,38,43]. Moreover, given that loop anchor regions are highly conserved, mutations in these regions may have significant effects such as loss of insulator function due to loop disruption. Here, we applied InsuLock on scATAC-seq peaks to identify non-coding variants that alter the 3D genome organization at the single-cell resolution in the human brain.

**Figure 6:**
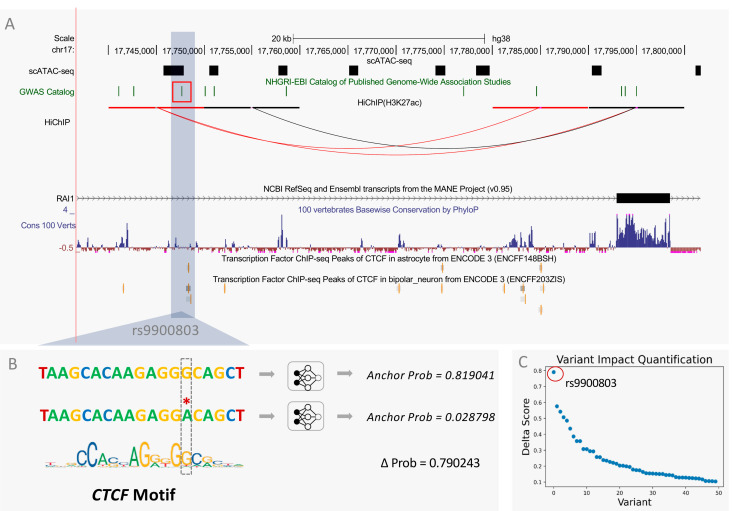
Total nucleotide InsuLock’s variant impact quantification module that predicts the effect of SNVs on the insulator sites. (**A**) Brain scATAC-seq, GWAS, HiChIP, NCBI RefSeq, 100 vertebrates PhyloP conservation, *CTCF* ChIP-seq peak in astrocytes, and bipolar neuron track on UCSC genome browser. (**B**) Predicted anchor probability of wild-type and mutant sequence. (**C**) Delta scores of GWAS SNPs associated with Schizophrenia.

Specifically, we used InsuLock to calculate the anchor probability of both WT and mutant sequences in Schizophrenia-related SNPs and computed a delta score for each variant by subtracting the probability values of WT and mutant sequences. We found that rs9900803, an intronic variant present in chromosome 17 and accessible only in astrocytes and oligodendrocyte cells, caused significant anchor disruption at the site of mutation with a delta score of 0.79, possibly due to the loss of *CTCF* motif on the complementary strand. As shown in Figure 6A, the SNP is located ~40 kb upstream of the transcription start site of the *RAI1* gene and localizes around a *CTCF* ChIP-seq peak in astrocytes. HiChIP data also indicated the presence of a possible loop connecting the site of the SNP to a region ~10 kb upstream of the transcription start site of the *RAI1* gene, which contains highly conserved regions around its anchor, indicating the presence of regulatory elements. The high delta score indicates a potential insulator loop disruption due to rs9900803, leading to upregulation of the *RAI1* gene from the loss of insulator activity. Previous studies have shown that dysregulation of the *RAI1* gene in the brain leads to intellectual disabilities such as Smith–Magenis syndrome and Potocki–Lupski syndrome [46,47,48]. Specifically, one study showed that patients with schizophrenia and bipolar disorder had a significant increase in *RAI1* expression in their brains [49]. However, the underlying mechanisms through which the *RAI1* gene is overexpressed in patients with schizophrenia are still unknown. We hypothesize a possible insulator loop disrupting mechanism around the promoter region of the *RAI1* gene, potentially through disruption of a *CTCF* binding motif, leading to the overexpression of the *RAI1* gene.

## 4. Discussion

In this paper, we present a weakly supervised learning scheme called InsuLock with three key modules for (1) accurate *CTCF* mediated insulator loop anchor prediction; (2) precise boundary refinement on the base-pair resolution using weakly supervised learning; and (3) variant impact quantification within insulator loop anchor sites. For the binary classification step, we trained a deep convolution neural network that robustly identifies complex non-linear sequence patterns to make accurate predictions. We also showed that our post-hoc conjoined model outperformed existing state-of-the-art approaches that fail to consider the double-stranded nature of DNA sequences. In addition to the binary classification model, InsuLock’s method has a unique weakly supervised object detection module implemented via Grad-CAM, which provides the important sequence patterns utilized by the model to make predictions and refine coarse insular regions. In particular, we showed that the refined insulator annotations are highly enriched with known loop anchor motifs and are evolutionarily conserved across vertebrate species. Furthermore, InsuLock has a variant impact module that predicts the effect of mutations at insulator sites with single base-pair resolution.

For future directions, DNA sequences can be integrated with other epigenetic data such as DNA methylation and histone modification [50], since anchors of chromatin loops contain distinct epigenetic marks [51], that might further improve the insulator prediction task.

To summarize, we introduce a powerful deep learning-based tool that could be widely utilized for insulator prediction and discovery. With corresponding ATAC-seq data, InsuLock can predict insulators not only at the bulk tissue level but also at the single-cell resolution. Since chromatin conformation assays such as single-cell Hi-C are relatively expensive compared to single-cell ATAC-seq, computational models such as InsuLock that can help decode the 3D genome organization at the single-cell resolution greatly advance the understanding of gene regulation and interpret functional non-coding variants at the single-cell level.

## 5. Conclusions

We developed a deep learning method called InsuLock for the identification and characterization of chromatin insulators, and performed extensive benchmarking using publicly available data from ENCODE and demonstrated its high accuracy and sensitivity. We utilized InsuLock’s object detection module to identify cell-type specific transcription factor involvement in chromatin looping, and showed the InsuLock’s refined insulator annotations are highly conserved across species. We also applied our method to brain single-cell ATAC-seq data to quantify cell-type-specific impact scores of GWAS SNPs associated with schizophrenia.

## Data Availability

*Rad21* ChIA-PET, *CTCF* ChIP-seq and ATAC-seq data were obtained from ENCODE. Brain single-cell ATAC-seq and HiChIP data is available from [38]. The software for the analyses can be found at https://github.com/aicb-ZhangLabs/InsuLock (accessed on 29 January 2022).

## Supplementary Materials

The following supporting information can be downloaded at: https://www.mdpi.com/article/10.3390/genes13040621/s1, Figure S1: Leave-One-Chromosome-Out Cross Validation; Table S1: A detailed list of transcription factor (TF) motif’s enriched within InsuLock’s refined insulator annotations, identified using CentriMo; Table S2: Differential motif enrichment analyses to identify cell-type specific TF involvement in chromatin looping.
