# Supplementary material for: InsuLock: A Weakly Supervised Learning Approach for Accurate Insulator Prediction, and Variant Impact Quantification
Source: Genes (Basel). 2022 Mar 30;13(4):621. doi: 10.3390/genes13040621 (PMC9026820; doi:10.3390/genes13040621)
Supplement: Supplementary file 1 [file genes-13-00621-s001.zip › genes-1599020-supplementary.pdf]

Supplementary files to “InsuLock A Weakly Supervised Learning Approach for Accurate Insulator Prediction, And Variant Impact Quantification”

1. Detailed specifications of the architecture and hyperparameters of InsuLock’s binary classification model

InsuLock utilizes a Siamese Convolutional Neural Network model to make binary predictions. The model consists of three convolution layers, three max-pooling layers, and three fully-connected layers. The initial convolution filters are trained to capture important sequence patterns such as transcription factor binding motifs, and the max-pooling layers help in summarizing the information learned by convolution filters and in reducing the total model parameters. The output from these layers is fed into fully connected layers, that facilitate in making a sigmoid output.

Input Dimension: 2000x4 one-hot-encoded DNA matrix

Model Architecture

1. Convolution layer (160 channels, kernel size: 31, stride: 1)
2. Max pooling layer (kernel size: 2, stride: 2)
3. Convolution layer (160 channels, kernel size: 20, stride: 1)
4. Max pooling layer (kernel size: 2, stride: 2)
5. Convolution layer (160 channels, kernel size: 6, stride: 1)
6. Max pooling layer (kernel size: 8, stride: 6)
7. Fully Connected layers (neurons: 925)
8. Fully Connected layers (neurons: 925)
9. Sigmoid Output layer

Dropout regularization

- Layer6: 50%
- Layer7: 50%
- All other layers: 0%

Supplementary Figures

Figure S1. Leave-One-Chromosome-Out Cross Validation

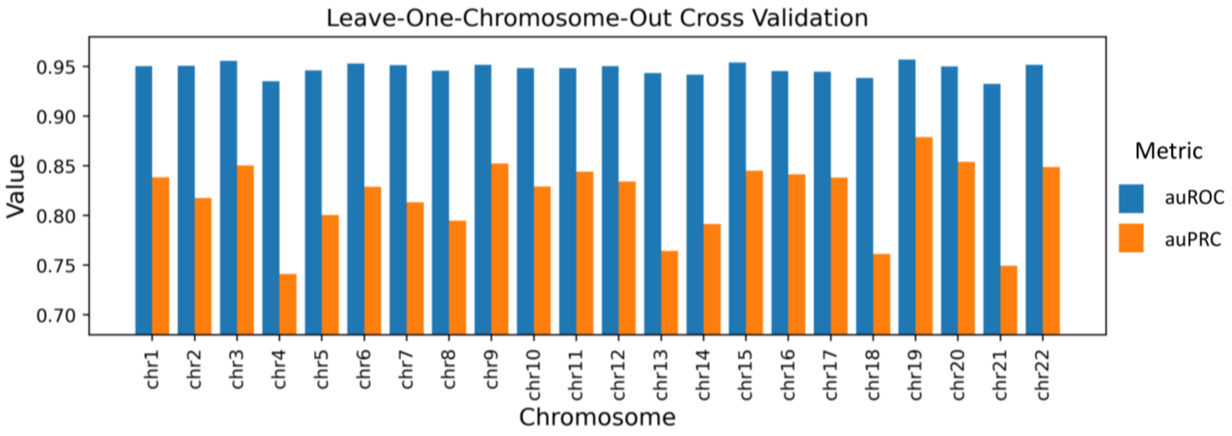

Supplementary Tables

Table S1. A detailed list of transcription factor (TF) motif's enriched within InsuLock's refined insulator annotations, identified using CentriMo

Rank	Motif_Name	p-value	adj_p-value	log_adj_p-value	E-value
1	CTCF	1.3e-1771	5.2e-1766	-4064.72	9.2e-1763
2	CTCFL	3.6e-1674	2.3e-1668	-3839.86	4.2e-1665
3	NR2C1	1.5e-875	3.6e-870	-2001.98	6.3e-867
4	ZIC4	7.1e-663	1.9e-657	-1512.17	3.3e-654
5	NR2C2	3.7e-537	8.1e-532	-1222.89	1.4e-528
6	ZIC3	6.4e-527	1.9e-521	-1199.02	3.3e-518
7	THAP1	4.0e-526	1.6e-520	-1196.9	2.8e-517
8	ZIC1	3.0e-507	5.4e-502	-1154.2	9.7e-499
9	YY2	3.7e-492	1.9e-486	-1118.42	3.4e-483
10	TFEC	9.4e-488	3.5e-482	-1108.59	6.2e-479
11	LMX1B	6.9e-483	3.2e-477	-1097.17	5.7e-474
12	NEUROD1	2.2e-471	1.2e-465	-1070.56	2.0e-462
13	HAND2	7.6e-468	2.3e-462	-1062.95	4.1e-459
14	ALX3	9.0e-439	4.1e-433	-995.6	7.4e-430
15	NEUROG2	3.9e-426	2.2e-420	-966.3	3.9e-417
16	UNCX	9.1e-417	4.1e-411	-944.96	7.2e-408
17	LMX1A	1.1e-389	5.1e-384	-882.57	9.0e-381
18	RAX2	2.0e-388	9.0e-383	-879.69	1.6e-379
19	TCF12	2.5e-386	1.5e-380	-874.56	2.7e-377
20	HIC2	1.2e-379	3.4e-374	-859.94	6.0e-371
21	ZNF141	1.5e-378	7.5e-373	-856.84	1.3e-369
22	MIXL1	3.8e-377	1.0e-371	-854.26	1.8e-368

23	ASCL1	7.7e-376	2.3e-370	-851.11	4.2e-367
24	ZIC5	3.2e-371	5.7e-366	-841.01	1.0e-362
25	USF1	1.8e-369	4.5e-364	-836.63	8.0e-361
26	ATOH7	4.6e-366	1.5e-360	-828.52	2.7e-357
27	E2F1	6.0e-359	1.7e-353	-812.31	2.9e-350
28	VSX1	2.1e-355	9.9e-350	-803.61	1.8e-346
29	MYC	1.2e-347	6.1e-342	-785.68	1.1e-338
30	GSX2	1.9e-346	5.3e-341	-783.51	9.5e-338
31	GSC2	2.4e-339	7.0e-334	-767.12	1.2e-330
32	FIGLA	4.5e-338	1.4e-332	-764.11	2.5e-329
33	E2F2	5.8e-333	1.3e-327	-752.69	2.3e-324
34	VAX1	1.9e-327	9.1e-322	-739.23	1.6e-318
35	TCF21	2.1e-324	6.4e-319	-732.67	1.1e-315
36	PRRX1	4.5e-324	1.0e-318	-732.19	1.8e-315
37	TCF4	1.6e-322	9.4e-317	-727.68	1.7e-313
38	TBX5	4.4e-321	1.1e-315	-725.26	1.9e-312
39	E2F4	2.5e-320	5.1e-315	-723.68	9.1e-312
40	MYCN	4.2e-317	1.6e-311	-715.6	2.90E-308
41	TBX3	5.4e-311	1.60E-305	-701.81	2.90E-302
42	NKX6-2	6.9e-310	1.70E-304	-699.43	3.10E-301
43	ZNF100	0.00E+00	6.10E-304	-698.18	1.10E-300
44	BARHL1	1.90E-298	5.20E-293	-673.01	9.20E-290
45	HOXA10	4.00E-298	1.30E-292	-672.08	2.30E-289
46	HOXB2	6.10E-298	1.80E-292	-671.77	3.20E-289
47	POU5F1B	1.00E-297	4.60E-292	-670.83	8.20E-289
48	DLX6	1.90E-297	4.90E-292	-670.77	8.70E-289
49	KLF2	3.90E-296	1.20E-290	-667.55	2.20E-287
50	VAX2	4.20E-291	2.00E-285	-655.54	3.60E-282
51	EBF1	6.70E-290	1.70E-284	-653.41	3.00E-281
52	ESX1	1.40E-288	5.10E-283	-650	9.00E-280
53	VSX2	3.40E-285	1.40E-279	-642.1	2.50E-276
54	POU5F1	9.90E-285	6.40E-279	-640.57	1.10E-275
55	NFIX	5.70E-284	1.50E-278	-639.73	2.60E-275
56	GSX1	1.30E-280	4.20E-275	-631.77	7.50E-272
57	MEOX2	1.20E-277	6.10E-272	-624.49	1.10E-268
58	PAX4	1.00E-276	2.50E-271	-623.1	4.40E-268
59	ZNF425	1.10E-272	5.80E-267	-613.03	1.00E-263
60	OTX2	2.50E-269	5.20E-264	-606.23	9.20E-261
61	POU3F1	1.30E-268	5.30E-263	-603.92	9.40E-260
62	TCF3	1.90E-265	1.20E-259	-596.17	2.20E-256
63	BHLHA15	1.20E-264	4.00E-259	-594.99	7.10E-256
64	RAX	1.40E-264	4.50E-259	-594.87	8.00E-256
65	TEAD4	1.80E-262	6.10E-257	-589.95	1.10E-253
66	GATA6	1.60E-255	8.30E-250	-573.53	1.50E-246
67	MEF2A	4.80E-255	2.20E-249	-572.55	3.90E-246

68	PITX3	3.40E-252	1.00E-246	-566.41	1.80E-243
69	GATA1	1.00E-251	5.80E-246	-564.68	1.00E-242
70	ZEB1	1.70E-248	4.30E-243	-558.06	7.70E-240
71	LHX9	3.60E-245	1.10E-239	-550.24	1.90E-236
72	POU4F1	9.70E-244	4.00E-238	-546.62	7.10E-235
73	POU3F3	1.90E-240	7.50E-235	-539.1	1.30E-231
74	TBX15	1.30E-238	1.30E-233	-536.21	2.40E-230
75	POU2F1	3.10E-235	1.40E-229	-526.97	2.40E-226
76	PBX2	1.60E-233	9.40E-228	-522.75	1.70E-224
77	HOXB3	3.60E-233	1.10E-227	-522.63	1.90E-224
78	LBX2	3.50E-232	1.10E-226	-520.33	1.90E-223
79	BARHL2	9.80E-231	2.80E-225	-517.06	4.90E-222
80	TBX4	3.30E-230	6.10E-225	-516.28	1.10E-221
81	ESRRA	9.00E-229	2.70E-223	-512.49	4.80E-220
82	GATA4	5.40E-228	3.10E-222	-510.03	5.60E-219
83	ZNF519	1.20E-227	4.00E-222	-509.78	7.20E-219
84	NKX6-1	3.20E-225	8.10E-220	-504.48	1.40E-216
85	LHX1	7.70E-224	1.80E-218	-501.36	3.20E-215
86	PHOX2B	6.00E-223	2.20E-217	-498.87	3.90E-214
87	MXI1	6.40E-223	2.50E-217	-498.73	4.50E-214
88	TCF7	1.30E-222	7.20E-217	-497.69	1.30E-213
89	TWIST1	1.90E-220	9.50E-215	-492.8	1.70E-211
90	ZNF296	4.20E-219	1.60E-213	-489.98	2.80E-210
91	NEUROD2	4.90E-215	1.50E-209	-480.86	2.60E-206
92	KLF6	4.20E-214	1.30E-208	-478.67	2.30E-205
93	HOXB13	2.40E-212	1.10E-206	-474.21	2.00E-203
94	MEIS2	3.50E-212	1.60E-206	-473.88	2.80E-203
95	SP9	9.70E-212	2.90E-206	-473.27	5.10E-203
96	TFE3	1.10E-210	3.00E-205	-470.95	5.20E-202
97	HOXA2	2.40E-209	7.00E-204	-467.78	1.20E-200
98	TBX6	3.30E-206	9.20E-201	-460.6	1.60E-197
99	GCM1	4.70E-204	1.10E-198	-455.79	2.00E-195
100	GATA2	3.50E-201	1.50E-195	-448.57	2.70E-192
101	ESR2	1.80E-200	4.70E-195	-447.47	8.30E-192
102	CDX1	6.00E-199	1.90E-193	-443.77	3.30E-190
103	HMBOX1	1.10E-197	3.60E-192	-440.8	6.50E-189
104	TEAD1	9.60E-196	3.30E-190	-436.29	5.90E-187
105	ZBTB7C	2.00E-195	5.90E-190	-435.71	1.10E-186
106	TFEB	7.50E-194	2.00E-188	-432.2	3.50E-185
107	POU4F3	3.90E-193	1.40E-187	-430.21	2.60E-184
108	MEIS3	7.80E-193	1.60E-187	-430.11	2.80E-184
109	PHOX2A	5.50E-190	1.60E-184	-423.2	2.90E-181
110	MSX1	6.80E-190	1.60E-184	-423.18	2.90E-181
111	FOXD2	1.80E-189	2.90E-184	-422.63	5.10E-181
112	BARX2	6.40E-188	1.70E-182	-418.56	2.90E-179

113	NOTO	1.10E-186	3.30E-181	-415.58	5.80E-178
114	POU2F3	2.20E-185	9.20E-180	-412.25	1.60E-176
115	TEAD2	3.70E-184	2.00E-178	-409.15	3.60E-175
116	ZBTB40	5.50E-184	2.80E-178	-408.83	5.00E-175
117	FOXK2	4.90E-182	3.50E-176	-404	6.20E-173
118	CDX2	1.50E-180	8.30E-175	-400.83	1.50E-171
119	DPF1	8.60E-180	3.40E-174	-399.44	6.00E-171
120	SOX18	5.70E-179	1.70E-173	-397.84	3.00E-170
121	OTX1	4.30E-178	7.30E-173	-396.36	1.30E-169
122	SNAI1	3.30E-176	9.00E-171	-391.55	1.60E-167
123	HOXA6	1.40E-175	3.90E-170	-390.09	6.90E-167
124	RBPJ	3.60E-175	9.80E-170	-389.16	1.70E-166
125	SCRT1	2.40E-174	9.30E-169	-386.91	1.60E-165
126	FOXP1	3.70E-174	2.70E-168	-385.85	4.80E-165
127	EN1	2.80E-172	6.70E-167	-382.64	1.20E-163
128	HOXA9	3.80E-172	1.10E-166	-382.16	1.90E-163
129	DRGX	2.10E-171	3.30E-166	-381.04	5.80E-163
130	NFIL3	9.90E-172	5.30E-166	-380.56	9.40E-163
131	EVX1	1.50E-168	4.20E-163	-373.88	7.50E-160
132	OLIG3	1.80E-166	5.40E-161	-369.02	9.60E-158
133	POU3F2	6.90E-166	2.00E-160	-367.74	3.50E-157
134	MSX2	2.00E-165	4.20E-160	-366.97	7.50E-157
135	GBX2	1.50E-164	3.50E-159	-364.87	6.20E-156
136	ATOH1	5.40E-164	8.10E-159	-364.02	1.40E-155
137	GSC	5.30E-163	1.50E-157	-361.11	2.60E-154
138	MEIS1	2.80E-162	1.40E-156	-358.87	2.50E-153
139	PRDM4	5.90E-162	3.90E-156	-357.85	6.90E-153
140	TFAP4	2.70E-161	8.80E-156	-357.03	1.60E-152
141	ZBTB7A	8.50E-161	2.70E-155	-355.91	4.80E-152
142	TBX1	5.60E-160	5.40E-155	-355.21	9.60E-152
143	ZNF597	1.10E-159	1.80E-154	-354	3.20E-151
144	E2F6	2.30E-159	1.20E-153	-352.1	2.20E-150
145	FOXA1	8.50E-158	4.70E-152	-348.44	8.40E-149
146	ISL2	1.30E-157	5.10E-152	-348.36	9.10E-149
147	EVX2	7.60E-157	2.10E-151	-346.97	3.70E-148
148	SCRT2	5.50E-157	2.10E-151	-346.93	3.80E-148
149	FOXP3	7.70E-156	1.20E-150	-345.23	2.10E-147
150	RUNX3	4.90E-156	1.30E-150	-345.15	2.30E-147
151	STAT3	5.30E-155	1.60E-149	-342.64	2.80E-146
152	MYOD1	2.90E-155	1.60E-149	-342.62	2.80E-146
153	GCM2	4.20E-154	1.20E-148	-340.58	2.20E-145
154	BSX	8.20E-154	1.90E-148	-340.15	3.30E-145
155	SNAI2	1.30E-153	3.10E-148	-339.66	5.50E-145
156	ZNF549	4.80E-153	3.00E-147	-337.4	5.20E-144
157	LHX2	8.80E-153	4.00E-147	-337.09	7.10E-144

158	RUNX2	2.50E-152	5.80E-147	-336.73	1.00E-143
159	ZNF652	1.70E-150	7.90E-145	-331.81	1.40E-141
160	YBX1	3.30E-150	8.90E-145	-331.68	1.60E-141
161	FOXA3	2.00E-148	1.40E-142	-326.6	2.60E-139
162	HOXC10	4.50E-145	1.30E-139	-319.82	2.30E-136
163	MSC	9.40E-145	2.10E-139	-319.33	3.70E-136
164	FOXJ2::ELF1	3.10E-144	8.00E-139	-317.98	1.40E-135
165	ZKSCAN1	1.20E-143	2.20E-138	-316.97	3.90E-135
166	MEF2C	2.20E-143	5.70E-138	-316.02	1.00E-134
167	ZNF677	4.80E-139	1.70E-133	-305.74	2.90E-130
168	ZBTB42	4.30E-139	2.30E-133	-305.41	4.10E-130
169	USF2	1.80E-137	2.80E-132	-302.92	4.90E-129
170	POU3F4	1.80E-137	6.00E-132	-302.14	1.10E-128
171	BATF	1.90E-137	1.10E-131	-301.53	2.00E-128
172	ELF5	2.50E-136	7.60E-131	-299.61	1.40E-127
173	SOX8	4.70E-136	1.50E-130	-298.95	2.60E-127
174	ZNF134	2.80E-135	5.00E-130	-297.72	8.90E-127
175	POU6F2	4.20E-134	1.20E-128	-294.54	2.20E-125
176	FOXC1	4.60E-134	1.40E-128	-294.39	2.50E-125
177	POU1F1	2.30E-133	5.20E-128	-293.08	9.30E-125
178	ZNF384	7.50E-133	1.80E-127	-291.82	3.20E-124
179	ETV5	9.50E-133	2.30E-127	-291.58	4.20E-124
180	FOXA2	1.50E-132	9.30E-127	-290.19	1.70E-123
181	HESX1	4.90E-132	1.40E-126	-289.79	2.50E-123
182	MAX	7.20E-131	1.90E-125	-287.21	3.30E-122
183	BATF3	6.60E-131	3.30E-125	-286.62	5.90E-122
184	PRDM1	1.10E-130	6.30E-125	-285.98	1.10E-121
185	SIX1	4.40E-130	2.40E-124	-284.63	4.30E-121
186	TFAP2A	2.30E-129	6.30E-124	-283.67	1.10E-120
187	BATF::JUN	8.00E-129	4.60E-123	-281.7	8.10E-120
188	ZNF28	2.90E-128	6.40E-123	-281.37	1.10E-119
189	MGA	6.50E-127	1.20E-121	-278.41	2.20E-118
190	GLI2	6.50E-127	1.60E-121	-278.15	2.80E-118
191	HOXA13	6.30E-127	2.30E-121	-277.76	4.20E-118
192	MEOX1	2.20E-126	6.90E-121	-276.68	1.20E-117
193	GRHL2	3.10E-126	1.30E-120	-276.06	2.30E-117
194	FOXB1	4.90E-125	1.50E-119	-273.6	2.70E-116
195	FOXC2	2.90E-124	1.40E-118	-271.37	2.50E-115
196	EGR1	7.10E-124	3.20E-118	-270.53	5.70E-115
197	GLIS2	2.20E-123	3.80E-118	-270.37	6.70E-115
198	ETV7	1.30E-121	3.80E-116	-265.77	6.70E-113
199	GBX1	4.10E-121	1.20E-115	-264.59	2.20E-112
200	IKZF1	9.40E-120	5.40E-114	-260.8	9.70E-111
201	KLF3	8.50E-119	2.00E-113	-259.49	3.60E-110
202	ARGFX	2.20E-118	5.60E-113	-258.47	9.90E-110

203	ETV4	3.90E-118	1.00E-112	-257.84	1.90E-109
204	MLXIPL	1.20E-117	3.00E-112	-256.79	5.40E-109
205	STAT1	2.80E-117	1.10E-111	-255.48	2.00E-108
206	ETV1	7.90E-116	2.00E-110	-252.59	3.50E-107
207	ZBTB20	1.60E-115	3.70E-110	-251.99	6.50E-107
208	ZFP42	1.20E-113	2.50E-108	-247.76	4.40E-105
209	ZNF766	6.40E-114	2.70E-108	-247.7	4.70E-105
210	POU6F1	1.30E-112	4.00E-107	-244.99	7.10E-104
211	HOXA7	2.60E-112	6.10E-107	-244.57	1.10E-103
212	OSR2	1.50E-112	9.70E-107	-244.1	1.70E-103
213	ISX	7.60E-112	1.00E-106	-244.06	1.80E-103
214	SNAI3	7.70E-112	1.80E-106	-243.5	3.20E-103
215	CREB3L4	6.00E-111	1.40E-105	-241.42	2.50E-102
216	MYB	7.20E-111	1.80E-105	-241.2	3.10E-102
217	HEY1	2.50E-109	5.40E-104	-237.78	9.60E-101
218	PROP1	3.80E-109	1.20E-103	-237.02	2.10E-100
219	BHLHE22	1.40E-108	1.50E-103	-236.79	2.60E-100
220	MSGN1	6.20E-109	1.80E-103	-236.58	3.20E-100
221	HOXD13	1.10E-107	3.50E-102	-233.61	6.20E-99
222	ZNF680	1.70E-107	5.70E-102	-233.12	1.00E-98
223	KLF11	2.90E-105	8.10E-100	-228.17	1.40E-96
224	ZNF133	2.90E-103	8.30E-98	-223.53	1.50E-94
225	HEY2	1.10E-102	2.70E-97	-222.37	4.80E-94
226	MYOG	3.90E-102	2.10E-96	-220.3	3.70E-93
227	ZFP91	7.70E-102	2.80E-96	-220	5.10E-93
228	EN2	1.60E-101	5.00E-96	-219.44	8.80E-93
229	BARX1	3.20E-100	5.10E-95	-217.11	9.10E-92
230	PITX2	4.80E-100	5.30E-95	-217.08	9.30E-92
231	ELK3	4.20E-100	9.60E-95	-216.48	1.70E-91
232	ZNF468	6.40E-100	2.50E-94	-215.54	4.40E-91
233	ZNF75D	1.20E-99	2.80E-94	-215.43	4.90E-91
234	ZNF707	1.60E-99	4.00E-94	-215.06	7.10E-91
235	ZNF148	2.60E-99	1.10E-93	-214.01	2.00E-90
236	NHLH1	2.00E-97	5.30E-92	-210.17	9.40E-89
237	MYNN	1.70E-96	7.00E-91	-207.59	1.20E-87
238	HOXC11	1.60E-95	4.40E-90	-205.75	7.80E-87
239	ERG	8.00E-95	1.70E-89	-204.41	3.00E-86
240	ZNF548	8.10E-95	2.40E-89	-204.04	4.30E-86
241	ETV6	1.10E-93	2.50E-88	-201.71	4.40E-85
242	DPRX	3.60E-92	1.00E-86	-198.02	1.80E-83
243	ZNF136	4.20E-89	1.20E-83	-190.94	2.10E-80
244	MNX1	1.00E-88	3.80E-83	-189.78	6.70E-80
245	MNT	1.80E-88	5.30E-83	-189.45	9.40E-80
246	SPIC	2.70E-88	7.10E-83	-189.16	1.30E-79
247	HOXD9	6.20E-88	1.60E-82	-188.32	2.90E-79

248	SOX2	9.40E-87	5.80E-81	-184.75	1.00E-77
249	ZNF506	2.00E-86	7.20E-81	-184.53	1.30E-77
250	KLF10	7.10E-86	1.90E-80	-183.57	3.40E-77
251	ONECUT1	2.30E-85	6.50E-80	-182.34	1.10E-76
252	CREB3L1	4.90E-85	6.70E-80	-182.31	1.20E-76
253	RFX7	5.90E-85	2.10E-79	-181.16	3.70E-76
254	ZNF534	9.90E-84	5.10E-78	-177.96	9.10E-75
255	HOXB6	3.20E-83	7.80E-78	-177.55	1.40E-74
256	SP2	7.60E-83	2.10E-77	-176.54	3.80E-74
257	TBX18	9.60E-83	3.10E-77	-176.18	5.40E-74
258	PRRX2	5.70E-81	5.20E-76	-173.34	9.30E-73
259	JUNB	1.80E-81	7.80E-76	-172.95	1.40E-72
260	TCF7L2	1.70E-79	3.80E-74	-169.05	6.80E-71
261	SOX10	8.50E-80	4.20E-74	-168.95	7.50E-71
262	ETV3	1.60E-79	5.20E-74	-168.75	9.20E-71
263	CUX1	2.30E-79	8.40E-74	-168.26	1.50E-70
264	MYF5	7.00E-79	3.40E-73	-166.88	5.90E-70
265	FERD3L	1.20E-77	1.90E-72	-165.13	3.40E-69
266	RELB	2.60E-77	1.50E-71	-163.07	2.70E-68
267	SP3	2.50E-77	1.60E-71	-163.03	2.80E-68
268	FOXK1	1.10E-76	5.30E-71	-161.82	9.30E-68
269	FOXO4	1.10E-75	9.60E-71	-161.22	1.70E-67
270	HOXA4	3.90E-76	9.80E-71	-161.2	1.70E-67
271	ETV5::FOXO1	6.30E-76	2.10E-70	-160.43	3.80E-67
272	RHOXF1	2.20E-75	4.60E-70	-159.67	8.10E-67
273	OLIG1	5.50E-75	1.00E-69	-158.86	1.80E-66
274	CUX2	4.80E-75	1.70E-69	-158.35	3.00E-66
275	SPI1	9.40E-75	2.30E-69	-158.04	4.10E-66
276	HOXC8	1.40E-74	3.20E-69	-157.72	5.70E-66
277	HOXC13	1.50E-73	3.70E-68	-155.27	6.50E-65
278	DUXA	1.00E-72	2.70E-67	-153.27	4.80E-64
279	LHX6	1.90E-72	5.20E-67	-152.62	9.30E-64
280	FEV	2.90E-72	7.10E-67	-152.31	1.30E-63
281	EBF3	2.00E-72	1.00E-66	-151.96	1.80E-63
282	KLF15	9.80E-72	2.20E-66	-151.19	3.90E-63
283	ZFP1	1.00E-71	2.80E-66	-150.95	4.90E-63
284	FLI1	1.00E-69	1.60E-64	-146.87	2.90E-61
285	HNF1A	1.50E-69	3.80E-64	-146.02	6.80E-61
286	EMX2	6.40E-69	1.70E-63	-144.54	3.00E-60
287	HOXC4	2.10E-68	3.00E-63	-143.95	5.40E-60
288	NFYC	9.50E-69	5.30E-63	-143.4	9.40E-60
289	FEZF1	1.00E-68	6.40E-63	-143.21	1.10E-59
290	HNF1B	6.30E-68	1.50E-62	-142.34	2.70E-59
291	JUND	4.10E-68	1.70E-62	-142.24	3.00E-59
292	OLIG2	1.20E-67	3.20E-62	-141.6	5.60E-59

293	HOXD11	1.10E-67	3.20E-62	-141.6	5.70E-59
294	VENTX	1.40E-67	3.50E-62	-141.52	6.20E-59
295	TFAP2C	1.40E-67	6.60E-62	-140.88	1.20E-58
296	STAT1::STAT2	3.70E-67	8.10E-62	-140.66	1.40E-58
297	ZBTB12	4.30E-67	2.60E-61	-139.49	4.70E-58
298	SREBF2	2.30E-66	3.60E-61	-139.17	6.40E-58
299	MAFF	5.70E-66	1.20E-60	-137.98	2.10E-57
300	ATF6B	1.40E-65	2.20E-60	-137.38	3.90E-57
301	FOSL1	2.10E-65	8.80E-60	-135.99	1.60E-56
302	YY1	5.10E-65	1.20E-59	-135.68	2.10E-56
303	NFATC4	6.40E-65	1.80E-59	-135.24	3.30E-56
304	PDX1	5.00E-64	6.10E-59	-134.04	1.10E-55
305	ZFP69	3.80E-64	7.60E-59	-133.83	1.30E-55
306	ZNF596	1.80E-64	8.60E-59	-133.7	1.50E-55
307	SIX2	3.90E-64	1.40E-58	-133.23	2.40E-55
308	TFAP2B	8.40E-64	2.40E-58	-132.7	4.20E-55
309	MAZ	6.90E-63	1.70E-57	-130.73	3.00E-54
310	ERF::FOXI1	2.80E-62	7.30E-57	-129.26	1.30E-53
311	MAFK	3.70E-61	8.90E-56	-126.75	1.60E-52
312	CEBPD	5.10E-61	2.40E-55	-125.75	4.30E-52
313	HNF4A	4.90E-61	2.90E-55	-125.59	5.10E-52
314	ZNF518A	6.50E-60	1.00E-54	-124.33	1.80E-51
315	ZNF92	2.50E-60	1.00E-54	-124.3	1.80E-51

Table S2. Differential motif enrichment analyses to identify cell-type specific TF involvement in chromatin-looping

Cell Line	Transcription Factor
GM12878	BATF::JUN
	E2F1::HES7
	GLIS2
	TFAP2C
	ETV2::FOXI1
	ZNF429
H1	ESR1
	ZNF317
	HOXD3
	ZNF765
	NR1I3
	HOXB7
	ESR2
K562	HOXD10
	ZNF254
	MEIS1
MCF7	NFYB
	E2F3::ONECUT2
